# RECRP: An Underwater Reliable Energy-Efficient Cross-Layer Routing Protocol †

**DOI:** 10.3390/s18124148

**Published:** 2018-11-26

**Authors:** Jun Liu, Meiming Yu, Xingwang Wang, Yuanyuan Liu, Xiaohui Wei, Junhong Cui

**Affiliations:** 1 College of Computer Science and Technology, Jilin University, Changchun 130012, China; liujun1509@jlu.edu.cn (J.L.); yumm5513@mails.jlu.edu.cn (M.Y.); liuyuanyuan16@mails.jlu.edu.cn (Y.L.); weixh@jlu.edu.cn (X.W.); junhong_cui@jlu.edu.cn (J.C.); 2 State Key Laboratory of Robotics, Shenyang Institute of Automation, Chinese Academy of Sciences, Shenyang 110016, China

**Keywords:** underwater wireless sensor networks (UWSNs), cross-layer, routing protocol

## Abstract

A reliable energy-efficient routing protocol plays a key role in underwater data transmission. In the face of acoustic communication challenges in underwater wireless sensor networks (UWSNs), including long propagation delay, topology change, limited energy, and communication voids, we propose RECRP, a Reliable Energy-efficient Cross-layer Routing Protocol to achieve high data delivery rate. RECRP is a location-free single-copy protocol. The information of the physical layer such as Doppler scale shift measurement, Received Signal Strength Indication (RSSI), etc. are adopted to estimate the distance, thus no extra hardware is needed for localization. Moreover, the overhead introduced by redundant packets is avoided with the single-copy mechanism. To improve the two-hop packet delivery rate and balance energy consumption among adjacent nodes, an optimal max–min method is proposed that dynamically controls transmission power and channel frequency. Furthermore, a surface to bottom routing establishment method is also adopted to handle communication voids. Compared with depth-based routing (DBR) and hop-by-hop vector-based forwarding (HH-VBF), RECRP is more energy-efficient with a higher delivery rate.

## 1. Introduction

**Figure 1:**
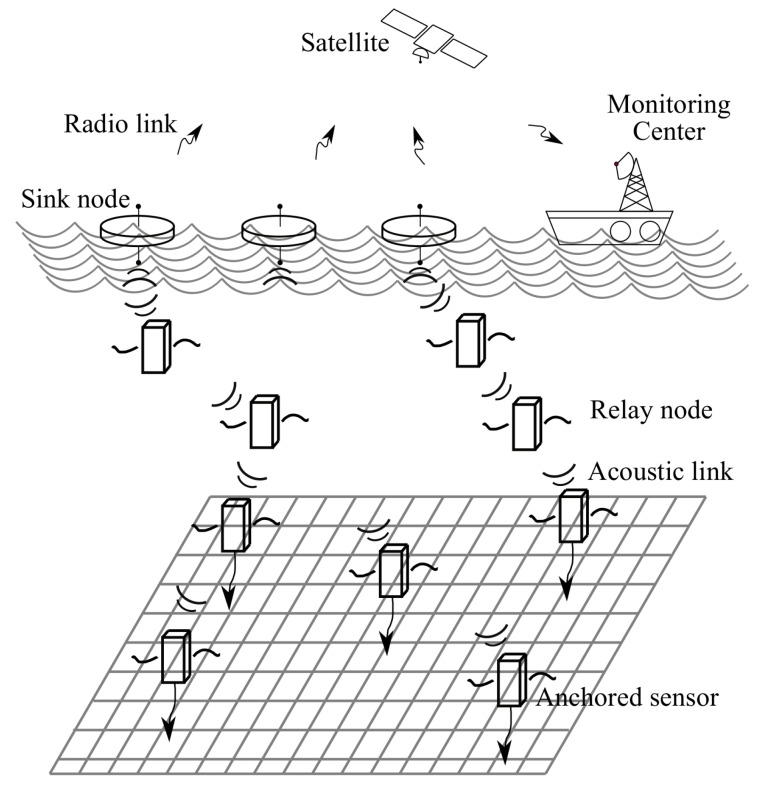
Target scenario.

Underwater wireless sensor networks (UWSNs) are becoming increasingly popular in applications such as real-time ocean monitoring, submarine tracking, offshore exploration, pollution monitoring, and hydrological environment protection. There are three wireless communication technologies in underwater environments, including electromagnetic, optical, and acoustic waves. With the exception of acoustic waves, the effective communication range is no more than 100 m because of the serious attenuation [1]. Since the acoustic wave is the only energy form that can propagate for a long distance in UWSNs, wireless acoustic communication is currently the dominant data delivery method for numerous underwater applications. In such applications shown in Figure 1, sensor nodes are placed at different depths to collect and forward aquatic information to sink nodes through acoustic links. Then, sink nodes transmit data to the monitoring center through radio links.

**Figure 2:**
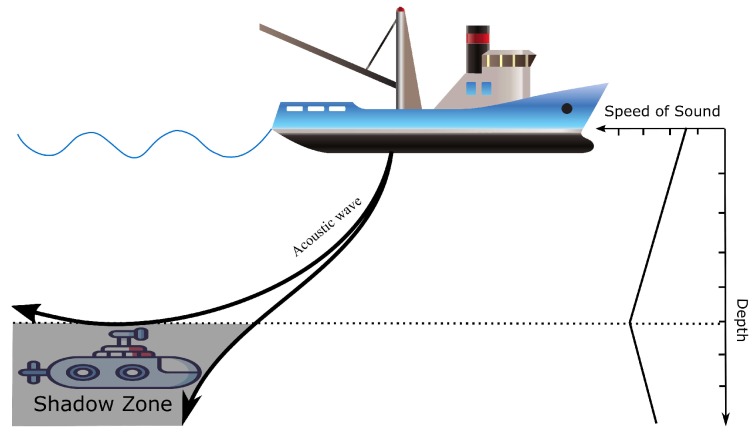
Generation of the shadow zone.

However, underwater acoustic communications also have limitations such as long propagation delay, limited energy consumption, limited bandwidth, complex ambient noise, and complex path loss which cause great trouble for UWSNs’ protocol design [2,3,4,5]. The propagation speed of sound in underwater environments is around 1500 m/s, which is five orders of magnitude slower than the speed of RF. As shown in Figure 2, sound velocity changes along with the factors such as water temperature, depth, density, and salinity, which means that acoustic wave propagation in underwater environments is nonlinear. The nonlinear propagation causes shadow zones where the transducers cannot receive from others, which leads to communication voids.

Moreover, the transmission power required by underwater acoustic transceivers is orders of magnitude higher than that of terrestrial devices. Since sensor nodes are often battery-powered and deployed deeply, the batteries are unavailable to replace when they are exhausted. Thus, the lifetime of an underwater node is severely restricted by its local power source [6]. Since the major concern for energy cost is transmitting rather than receiving, the overall energy consumption along the path to sink nodes must be carefully controlled. In addition, neighbor nodes near sink nodes are used frequently for data forwarding, which accelerates energy consumption. This results in an imbalance of energy consumption as well as premature exhaustion of node energy.

Terrestrial routing protocols have poor performance in underwater environments. Existing routing protocols proposed for terrestrial mobile and ad-hoc networks are usually divided into two categories: proactive and reactive. However, neither of these protocols is suitable for underwater environments. Proactive protocols require too much signaling overhead to establish end-to-end routing, especially when the network first establishes. Once the topology changes, the establishment and maintenance of the routing table causes huge network overhead. Constant movement of underwater nodes leads to frequent network topology changes, which aggravates the situation. Reactive routing protocols are suitable for dynamic environments, but they face large delays as they require source-initiated flooding for the route discovery process. Moreover, experiments show that reactive routing protocols perform poorly in asymmetric links (UWSNs). Thus, more and more researchers are devoted to the design of new routing protocols for UWSNs [7].

Greedy routing protocols are studied extensively to improve the packet delivery rate with minimum cost [8]. Due to high mobility of underwater nodes, greedy hop-by-hop routing is the most reliable method. Since this technique relies on a simple strategy that forwards packets to the locally optimal node with a strict positive progress towards the sink node, it does not work properly when data packets reach a node that has no neighbors with positive progress towards the sink node. This phenomenon might be caused by node mobility or the existence of shadow zones. This is the problem greedy routing protocols are generally faced with, known as the communication void problem.

In this paper, we propose a reliable energy-efficient cross-layer routing protocol called RECRP. RECRP is a location-free single-copy protocol, and does not rely on location and depth information. To improve two-hop packet delivery rate and balance energy among the network, we propose an optimal max–min method that dynamically controls transmission power and channel frequency. Since the Doppler scale shift can be measured from a single message exchange between two nodes, we adopt information from the physical layer such as Doppler scale shift measurement, Received Signal Strength Indicator (RSSI), etc. [9,10] to select next hop without the use of any additional hardware (e.g., depth probes, inertial sensors). Hence, RECRP reduces the equipment cost significantly.

The meaning of cross-layer routing is to use and adjust more underlying information in the routing protocol, such as using Doppler scale shift to estimate the relative speed between nodes, and adjusting the transmission power and frequency to improve the transmission efficiency. RECRP includes routing table updating and routing phases. The updating phase updates the routing table information. According to the routing table, the routing phase dynamically selects communication frequency, transmission power, and the next hop to achieve energy-efficient forwarding. We chose the minimal forwarding capability between the node *i* to its neighbor j(k,h…) and the node j(k,h…) to its next hop to determine the minimal forwarding capability $C_{j}$ ($C_{k}$, $C_{h}$, …) within two hops (shorter board in Barrel principle), then selected the maximal among all minimal forwarding capabilities ($C_{j}$, $C_{k}$, $C_{h}$, …) to balance the energy consumption.

The following are the contributions of this paper:

- We propose a location-free single-copy routing protocol, RECRP, to deal with the high energy consumption and imbalanced energy cost in underwater sensor networks. We use the mixed integer linear programming (MILP) model with a max–min method to formulate the underwater routing forwarding problem to achieve energy efficiency. The protocol also handles the communication void problem, which is a common problem in greedy routing protocols.
- We adopt the information of physical layer such as Doppler scale shift measurement and RSSI to estimate the relative velocity and distance without extra hardware to make RECRP suitable for many underwater scenarios.
- We conducted simulations to verify the performance of RECRP in a randomly deployed sparse network. Simulation results show that in terms of packet delivery rate, energy consumption, and end-to-end delay, RECRP performed better than depth-based routing (DBR) and hop-by-hop vector-based forwarding (HH-VBF).

The rest of the paper is organized as follows. Previous work on underwater energy-efficient routing is summarized in Section 2 . In Section 3 we describe RECRP in detail. Section 4 illustrates simulation results. Finally, Section 5 concludes the paper.

## 2. Related Work

The first important issue of an underwater sensor network is to improve its energy efficiency to prolong the network lifetime. Energy-efficient underwater routing protocols typically use some special node information to reduce the energy consumption. They can generally be classified into the following three categories: location-based routing, depth-based routing, and path consumption-based routing.

**Location-based** routing protocols assume that each node knows its location and each packet contains the position of the transmitter, which may not be practical in floating oceans. Xie et al. present VBF [11], a vector-based protocol where the source to sink vector acts as a virtual pipeline with a pre-defined radius to reduce flooding packets for energy efficiency. Because it is difficult to define the pipe radius, VBF is difficult to deploy in UWSNs. HH-VBF [12] is an enhanced version of VBF where the source-to-sink vector is replaced by the per-hop vector. However, the small pipeline radius has lower performance in sparse networks. AHH-VBF [13] is an advanced version of HH-VBF that adaptively changes the radius and sends duplicate packets to improve reliability. Because of duplicate packets, there is a higher probability of collisions and energy consumption in AHH-VBF. GEDAR [14] and DCR [15] are two typical distributed topology control algorithms to avoid communication voids. They avoid communication voids by adjusting nodes’ locations. However, due to the large energy consumption of position adjustment, it is not energy-efficient. In a location-based routing protocol, the assumption that each node knows its location information in UWSNs is not necessarily true. Therefore, there are significant restrictions on the application scenario of location-based underwater routing protocols.

**Depth-based** routing protocols use depth information to route packets instead of full location coordinates. To acquire depth information, extra sensors are equipped which increases the cost of sensor nodes. DBR [16] was presented to forward packets to upper nodes. Since it uses a flooding method to route packets, DBR is not energy-efficient. DBMR [17] leverages a multi-hop transmission model to deliver packets to reduce the energy consumption. While protocols like DBR and DBMR cannot deal with communication voids, D-DBR [18] is an enhanced version of DBR that improves the time and energy costs. Since many nodes broadcast at the same time, D-DBR leads to a high probability of packet collisions.

**Path-consumption-based** routing protocols select the next hop by their considered route selection metric, including distance, hop count, and residual energy, to find the most energy-efficient path. ERP2R [19] forwards packets according to the physical distance from sink nodes and the residual energy. EEF [20] uses residual energy, depth, and distance from sink nodes to evaluate the cost. However, both EEF and ERP2R do not consider the effects of channel frequency and dynamic transmission power on the energy cost.

## 3. RECRP Description

We propose a reliable energy-efficient cross-layer underwater routing protocol, RECRP, to route packets to sink nodes. RECRP is a two-phase routing protocol, including an updating phase and a routing phase. The routing table in each node is built during the updating phase, and is updated in both updating and routing phases.

### 3.1. Routing Updating Phase

In RECRP, each sink node periodically broadcasts a routing update message to start the routing update phase. When a node receives a routing update message, it updates its routing table information and broadcasts a new route broadcast message. During the routing update phase, all nodes broadcast with maximum power and lower channel frequency to achieve the maximum communication range.

The update message contains packet type, node ID, node level, and residual energy. According to packet type, packets are divided into updating packets, data packets, and data response packets (acknowledgement packet, ACK). The node level is the minimum hop count to sink nodes, and the level of each sink node is 0.

Since each node needs to update its own routing table and broadcast a new updating message after receiving a routing update message, each node sends only one routing update message during the updating phase in order to avoid loops. In RECRP, since each node sends a routing update message in the next time slot after receiving the message, when a node receives the second updating message, it only records the node in the routing table.

Each node maintains a routing table as shown in Table 1. Each record contains the information used in the routing phase, such as the neighbor node ID, the distance to the neighbor node, the relative speed, and the residual energy. When a sensor node receives an updating message, data message, or ACK, it updates the corresponding entry in the routing table according to the received information. In order to adapt to the dynamic underwater network, each entry in the routing table corresponds to a timer for periodically clearing nodes that have not communicated recently. When the timer expires, the corresponding entry is removed from the routing table, which means that the node corresponding to the entry might move out of the communication range or become energy exhausted.

To obtain the distance between neighbor nodes, the combination of the path attenuation model, the received channel strength, and the transmission strength in data packets are utilized. The path attenuation model [21] for underwater acoustic communication with frequency *f* between two nodes at distance *l* is:

(1) $$A\left(l,f\right)=A_{0}l_{k}a{\left(f\right)}^{l},$$

where $A_{0}$ is a unit-normalizing constant, *k* is a propagation factor which represents the geometry of propagation, for spherical propagation *k* = 2, for cylindrical propagation *k* = 1 (here *k* = 1.5 [22]), and a(f) is the absorption coefficient. If expressed in dB, the underwater acoustic path loss is:

(2) $$10\log A\left(l,f\right)/A_{0}=k10\log l+l10\log a\left(f\right).$$

The first item on the right side of the formula indicates the propagation consumption, and the second item indicates the absorption of the medium. The absorption coefficient is often set empirically, and the absorption coefficient of the sound signal with frequency *f* in dB/km is defined by the Thorps formula [23]:

(3) $$10\log a\left(f\right)=0.11f^{2}/\left(1+f^{2}\right)+44f^{2}/\left(4100+f^{2}\right)+2.75*10^{-4}f^{2}+0.003.$$

The above formula is often used to represent signals with frequencies above a few hundred Hz. For low-frequency signals, the following form is often used:

(4) $$10\log a\left(f\right)=0.002+0.11f^{2}/\left(1+f^{2}\right)+0.011f^{2}.$$

**Figure 3:**
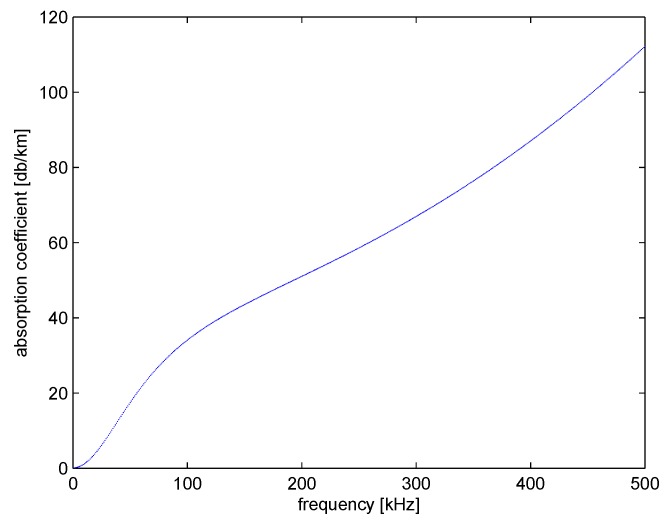
The relationship between absorption coefficient and frequency.

The relationship between absorption coefficient and frequency is shown in Figure 3.

Equation (2) describes the attenuation of the underwater acoustic signal without obstacles in the propagation path. If a hydro-acoustic signal of frequency *f* is transmitted with power *P* along an unblocked path, the received signal strength is P/A(l,f), so we can roughly estimate the distance between two nodes by the received signal strength.

The distance to the neighbor node is obtained from RSSI. Using RSSI to estimate distances for UWSNs needs to take into account multipath effects due to surface reflection, bottom reflection, and backscattering [24]. RSSI-based schemes can provide a ranging accuracy of a few meters [25]. Experiments reported in [26] have shown that multipath effects can be modeled as Rayleigh fading in shallow water. Therefore, RSSI is more suitable for deep water, with less reflection and refraction. For shallow water, we can use the peak value of signal intensity to estimate the distance.

The relationship between Doppler scale shift and relative velocity between any pair of nodes is v=a·c, where *v* is the relative velocity, *a* is the Doppler scale shift, and *c* is the sound speed. Relative velocity estimations based on Doppler can achieve a promised performance from the obtained data. For example, Mason et al. [27] reached 0.1 m/s deviation for node speed with a maximal velocity of 5 m/s (9.7 knot). We leveraged the Doppler measurement to estimate the relative velocity.

In underwater sensor networks, the network topology changes with nodes drifting in water currents. Therefore, the routing table needs to be updated frequently. However, frequent updates to the routing table requires a large number of control messages to be sent, which increases energy consumption. Considering the limited energy of the sensor node, the update frequency initiated by sink nodes should be based on the information on ocean currents. ACK is not required in the updating phase to save energy. It is assumed that sensor nodes are clock synchronized and that messages must be sent within a single time slot. In order to reduce packet collisions caused by multiple nodes sending messages at the same time, sensor nodes randomly select the transmission time in each time slot. Therefore, the time slot is much longer than the time required to send data messages.

### 3.2. Routing Phase

In the routing phase, collected data is sent to sink nodes through relay nodes. To ensure end-to-end transmission, it is necessary to ensure the successful transmission of each hop in the routing phase. When a node receives a packet, it sends an ACK to inform that the packet has been successfully received. If the sending node does not receive ACK from the receiving node within a certain period, the packet is sent repeatedly until ACK is received or retransmission times exceed the threshold.

In order to update path information in time, each ACK packet and data packet contain residual energy and transmission power information to inform neighboring nodes to update their corresponding routing table entries. When a node receives a packet, whether it is the next hop node or not, it always updates its routing table according to the information in the packet, and resets the corresponding timer.

In order to transmit data successfully and energy-efficiently, the probability of success in sending packets is estimated at different frequencies and powers during each transmission. First of all, we need to estimate the impact of noise on the underwater transmission.

Underwater ambient noise is mainly caused by four sources, including turbulence, shipping, waves, and thermal noise. We use $N_{t}\left(f\right)$, $N_{s}\left(f\right)$, $N_{w}\left(f\right)$, and $N_{th}\left(f\right)$ to represent these noises, respectively, where *f* is the underwater acoustic frequency used for packet transmission. The relationship of the four noises with frequency *f* can be described by the following formulae:

(5) $$\begin{array}{c} 10\log N_{t}\left(f\right)=17-30\log f, \end{array}$$

(6) $$\begin{array}{c} 10\log N_{s}\left(f\right)=40+20\left(s-0.5\right)+26\log f-60\log\left(f+0.03\right), \end{array}$$

(7) $$\begin{array}{c} 10\log N_{w}\left(f\right)=50+7.5w^{\left(1/2\right)}+20\log f-40\log f\left(f+0.4\right), \end{array}$$

(8) $$\begin{array}{c} 10\log N_{t}h\left(f\right)=-15+20\log f. \end{array}$$

Turbulent noise can only affect underwater acoustic signals with frequencies below 10 Hz. Shipping noise is the main noise between 10 and 100 Hz, and is described by shipping activity factor *s*. The value of *s* is between 0 and 1, and the smaller the value is, the less the shipping activity will be. Wave noise caused by sea breeze is the main noise between 100 Hz and 100 kHz. In the above formula, *w* is the sea breeze speed in m/s. Thermal noise is the main source of noise above 100 kHz.

**Figure 4:**
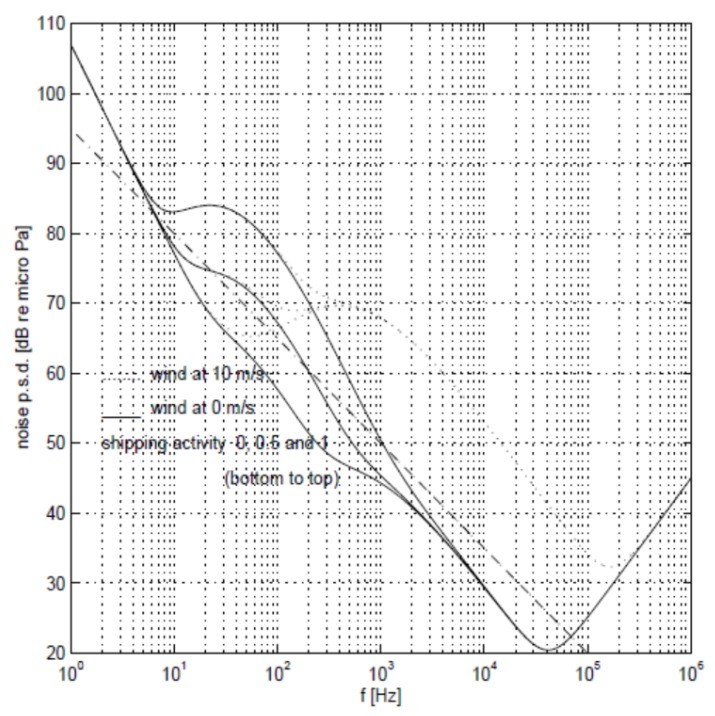
Schematic diagram of underwater noise variation with transmission frequency.

Figure 4 shows the relationship between transmission frequency and noise. The total ambient noise is [21,28,29]:

(9) $$N\left(f\right)=N_{t}\left(f\right)+N_{s}\left(f\right)+N_{w}\left(f\right)+N_{th}\left(f\right).$$

Given the attenuation A(l,f) and noise N(f), the signal-to-noise ratio (SNR) can be observed over a distance *l* at frequency *f* and power *P*:

(10) $$SNR\left(l,f,P\right)=\frac{P/A(l,f)}{N(f)}=\frac{P}{A(l,f)N(f)}.$$

The received packet can be correctly decoded when the SNR at the receiver is greater than the detection threshold. In addition to the SNR, the transmission of data packets should also account for the error probability of data transmission.

It is assumed that binary phase shift keying (BPSK) is used to modulate the underwater acoustic data. In BPSK, each symbol represents one bit, then the probability of an error of one bit of data over distance *l* with sending power *P* at frequency *f* is [30,31]:

(11) $$p_{e}\left(l,f,P\right)=\frac{1}{2}\left(1-\sqrt{\frac{SNR(l,f,P)}{1+SNR(l,f,P)}}\right).$$

For any two nodes with distance *l* on sending power *P* and communicating at frequency *f*, the probability of correctly delivering a packet with the size of *n* bits can be calculated as follows:

(12) $$p\left(l,f,P,n\right)={\left(1-p_{e}\left(l,f,P\right)\right)}^{n}.$$

Packet error rate can be reduced by an error correction code. Error correction codes (ECCs) are used for controlling errors in data over unreliable or noisy communication channels [32]. The central idea is that the sender encodes the message with a redundancy in the form of an ECC. ECCs include forward error correction (FEC) and automatic repeat request (ARQ). With FEC, the receiver corrects errors based on the received redundancy from the sender to minimize bit errors in the communication process. With ARQ, the sender is responsible for error correction by retransmitting unsuccessfully received packets to assure transmission reliability [33]. Since the error correction code is not the focus of this article, it is not considered in this paper.

The number of times that a packet will be sent at least once successfully is expected to be $\frac{1}{p(l,f,n,P)}$. Therefore, the expected energy consumption for the successful transmission of the packet is:

(13) $$P_{e}\left(l,f,n,P\right)=\frac{n}{\Delta f}\times \left\lceil \frac{1}{p(l,f,n,P)}\right\rceil ,$$

where Δf is the channel bandwidth. Since we use BPSK to modulate the underwater acoustic data, a symbol duration can be coarsely calculated as the inverse of the channel bandwidth. In this way, the time taken to send a packet with the actual duration is simply given by the number of bits in the packet, divided by the binary symbol duration of the BPSK waveform in use, so the time taken to send a packet with a data length of *n* is n/Δf.

The next hop of the packet at node *i* is determined by the node level, residual energy, residual energy of neighbor nodes *j*, and the distance evaluation between the current node and the neighbor node. These variables are denoted as $l_{i}$, $E_{i}$, $E_{j}$, and $L_{ij}$, respectively. In order to estimate the energy required to send a packet to the next hop, we need to calculate the distance between these two nodes. We use the distance between nodes *i* and *j*, $L_{ij}^{0}$, which is stored in the routing table, and the relative velocity between nodes *i* and *j*, $v_{ij}$, to estimate the distance between nodes *i* and *j*, $L_{ij}$. Assuming that the data updating time in the corresponding routing table is $t_{0}$, and the current time is *t*, then the distance between nodes *i* and *j* can be estimated as:

(14) $$L_{ij}=\alpha \left(L_{ij}^{0}+\left(t_{0}-t\right)v_{ij}\right),$$

where α is a constant parameter that exceeds 1 to guarantee that the evaluated distance is larger than the real one to overcome the inaccurate distance estimation introduced by RSSI-based distance estimation and Doppler-based velocity estimation.

We define the forwarding capability (nodes’ lifetime) of a node as the number of packets it can forward, and take it as a metric. In this problem, it is assumed that the energy consumption of sending an ACK is *K* times (with K<<1) the energy consumption of the transmitted data packet, and the energy consumption of the next hop forwarding the data packet is consistent with the energy consumption of the node sending the packet. The lifetime of the next hop node can be expressed as:

(15) $$\frac{\left(E_{i}-\left(1+K\right)P_{e}\left(l,f,n,P\right)\right)}{P_{e}\left(l,f,n,P\right)}\;,$$

where $E_{i}$ is the residual energy of node *i*, $E_{j}$ is the neighbor’s residual energy, and *l* is the distance to the neighbor. Then, the problem can be formulated as a MILP model:

(16) $$\begin{array}{c} \max\min\\ \;\{\frac{E_{i}-P_{e}\left(l,f,n,P\right)}{P_{e}\left(L_{ij},f,n,P\right)},\sum_{j}\frac{x_{ij}\left(E_{j}-\left(1+K\right)P_{e}\left(l,f,n,P\right)\right)}{P_{e}\left(l,f,n,P\right)}\}\\ \begin{array}{cc} s.t. & L_{P}\leq P\leq U_{P}\\ & L_{f}\leq f\leq U_{f}\\ & x_{i}H_{i}>x_{j}H_{j}\\ & \;\sum_{j\in neighbour(i)}x_{ij}=1\\ & x_{ij}\in \left[0,1\right] \end{array}, \end{array}$$

where $L_{P}$, $U_{P}$, $L_{f}$, and $U_{f}$ are lower and upper bounds of transmitting power and channel frequency, respectively. $H_{i}$ is the node hop, and $x_{ij}$ is a binary variable that indicates the next hop. If node *j* is the next hop, $x_{ij}=1$, otherwise, 0.

The significance of this optimization problem is to maximize the minimum forwarding capacity of the node and its neighbors after the message is forwarded. The next hop selection of the data packet is based on the information in the routing table, which is updated from ending (sink nodes) to beginning (sensor nodes). Therefore, communication voids can be avoided in RECRP.

## 4. Simulation

### 4.1. Simulation Setting

In order to verify the efficiency of RECRP, we simulated it using a Monte Carlo method. All numerical simulation results were obtained after 1000 calculations. In this simulation, relay nodes (ranging from 100 to 600) were randomly deployed in a 10 km × 10 km × 10 km area which made the network sparse. Fifty sensor nodes were fixedly deployed on the seafloor, and 10 sink nodes were randomly deployed on the ocean surface. The upper transmission power threshold was 90 dB re μPa, and the SNR threshold was 20 dB. The frequency was constrained within 0 to 35 kHz, which is available in the underwater acoustic channel. We also assumed that each node spent 1 W on sending and receiving a packet, which is caused by updating the routing table and other computation. The idle power was assumed to be 10 mW. The size of control packets and ACK packets were both 64 bits, and a data packet was 256 bits. BPSK modulation was used in this simulation, which means the bit rate was capped at 35 kbps. Then, each packet at least can be completely sent within 7.4 ms. We assumed that each sensor node sent sensed data every 1 min.

We compared RECRP with DBR and HH-VBF, and the frequency of the channel used by DBR and HH-VBF was fixed at 16 kHz, the transmission power was 90 dB re μPa, and the pipeline radius in HH-VBF was 1800 m.

DBR (depth-based routing) adopts a flooding routing mechanism and uses depth information for routing. When a node wants to send a packet, it will sense its current depth relative to the sink node and place this value in the packet header field and then broadcast it. Packets are forwarded when the depth is shallower than the sending node. HH-VBF (hop-by-hop vector-based forwarding) uses a similar concept of virtual routing pipe as used by VBF. VBF is a vector-based protocol where the source-to-sink vector acts as a virtual pipeline with a pre-defined radius to reduce flooding packets for energy efficiency. HH-VBF defines a per-hop virtual pipe for each forwarder.

In this work, a kinematic model [34] is applied to model the ocean current as follows:

(17) $$\left\{\begin{array}{c} v_{0|x}=k_{4}+k_{1}\lambda vsin\left(kk_{2}x\right)cos\left(kk_{3}y\right)+k_{1}\lambda cos\left(2kk_{1}t\right),\\ v_{0|y}=k_{5}-\lambda vcos\left(kk_{2}x\right)sin\left(kk_{3}y\right), \end{array}\right.$$

where $v_{0|x}$ and $v_{0|y}$ represent the velocity along the *x*-axis and *y*-axis, respectively. Parameters $k_{1}$, $k_{2}$, $k_{3}$, λ, and *v* characterize environmental factors such as the strength of tides and bathometry. $k_{4}$ and $k_{5}$ are random variables used to simulate the random factors. *k* is a coefficient applied to control the frequency of the changing direction. These variables are given as in Table 2, where N(A,B) is a normal distribution with the mean *A* and variance *B*.

The noise decays linearly in the logarithmic scale, and can be expressed as follows [21]:

(18) $$10\log N\left(f\right)=N_{1}-\eta \log f,$$

where $N_{1}$ was set to 95 and η was set to 15.

### 4.2. Results and Analysis

**Figure 5:**
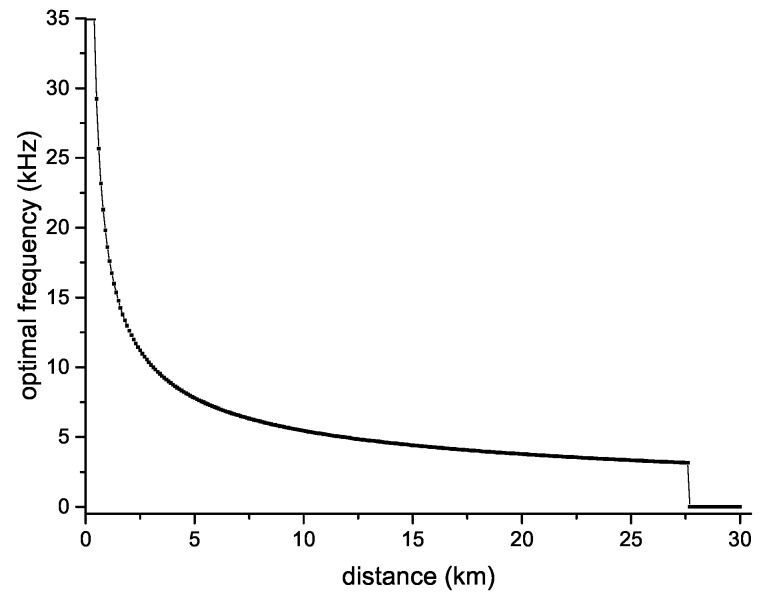
Optimal frequency with 90 dB re μPa transmission power.

Figure 5 shows the optimal transmission frequency between two nodes at different distances when the transmission power was 90 dB re μPa. The optimal transmission frequency here means that the expected energy consumption for successfully transmitting a data packet is the smallest. It can be seen that as the distance increased, the optimal transmission frequency decreased. When the distance exceeded 27 km, since the SNR was smaller than the threshold we set, the data could not be correctly decoded within the available frequency ranges, so there was no such optimal transmission frequency.

**Figure 6:**
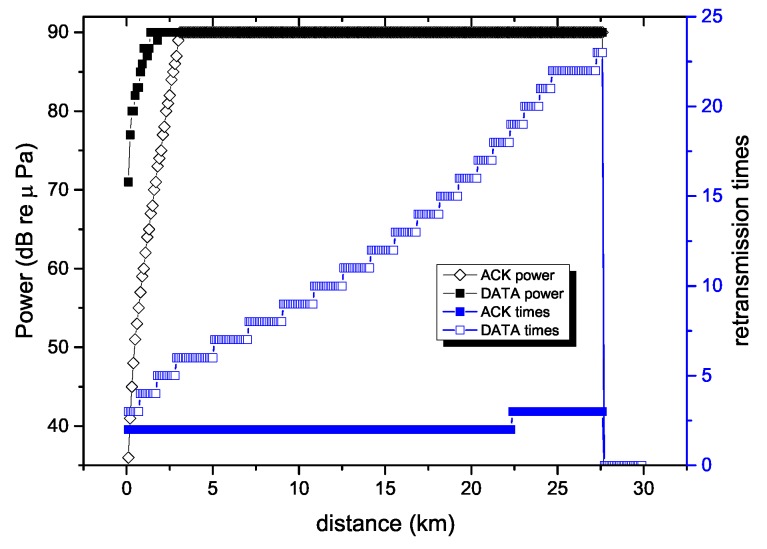
Transmission power and retransmission times with minimized energy cost. ACK: data response packet.

Figure 6 shows the transmission power and the expected retransmission times as a function of distance in order to ensure energy efficiency. It can be seen that as the transmission distance increased, the transmission power of the RECRP also increased, and the number of retransmissions of the data packet and the ACK packet increased as the distance increased. Since data messages are longer than ACK messages, the probability that the data message is correctly transmitted is less than that of ACK packets. Therefore, the retransmission of ACK packets was much less than that of data packets.

**Figure 7:**
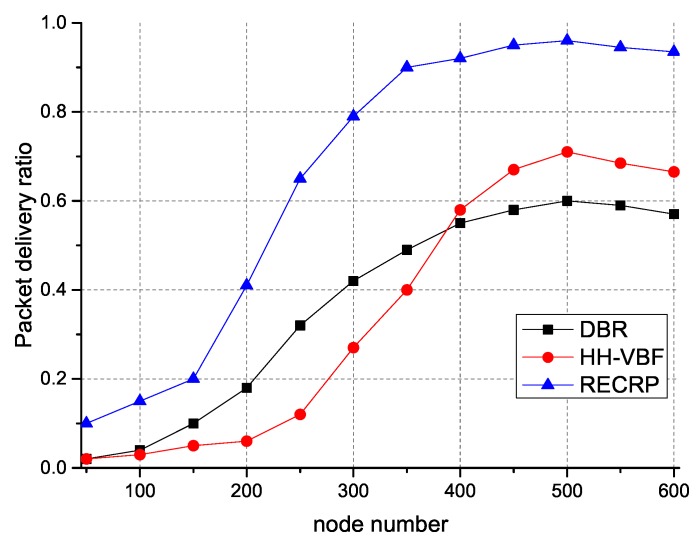
Delivery ratio with different numbers of nodes. DBR: depth-based routing; HH-VBF: hop-by-hop vector-based forwarding; RECRP: reliable energy-efficient cross-layer routing protocol.

As shown in Figure 7, it is easy to observe that RECRP had a higher delivery rate than DBR and HH-VBF. This is because we calculate relative velocity and distance through Doppler effect and RSSI, and dynamically select the appropriate power *P* and frequency *f* to choose the next hop. We can easily observe that the delivery rate got higher as the number of nodes increased. By setting a random sending time, RECRP achieves a lower probability of collisions. Additionally, we adopt a communication voids avoidance mechanism. Together with that, RECRP achieved a higher delivery rate than DBR and HH-VBF. DBR uses the global flooding mechanism, so the initial delivery rate was higher than HH-VBF, as the number of nodes increased, the collisions happened more frequently and the delivery rate became lower than HH-VBF.

**Figure 8:**
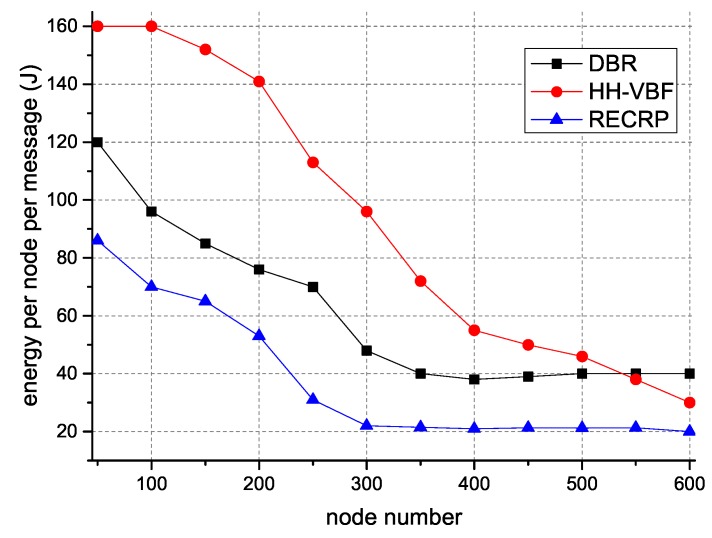
Energy consumption with different numbers of nodes.

Figure 8 illustrates energy consumption with different numbers of nodes. Its clear that the energy consumption per node of RECRP was much lower than DBR and HH-VBF. This is because we estimated the distance between nodes and selected the appropriate transmit power *P* and channel frequency *f*. The delivery rate is guaranteed, and the retransmission times were reduced. Our single-copy mechanism also avoids redundant packets to achieve the goal of prolonging the network lifetime. As the number of nodes increased, the retransmission times increased. The reason that the energy consumption was still smaller than DBR and HH-VBF is that RECRP has a lower probability of collisions and the transmission power *P* required for RECRP is relatively minimized. It is easy to observe that the energy per node per message of HH-VBF was higher than DBR. This is because the working mechanism of HH-VBF is to select nodes in the virtual pipeline. The candidate nodes are smaller than DBR, which adopts the flooding method, so the probability of successful forwarding becomes smaller, which leads to more retransmissions and larger total energy consumption.

**Figure 9:**
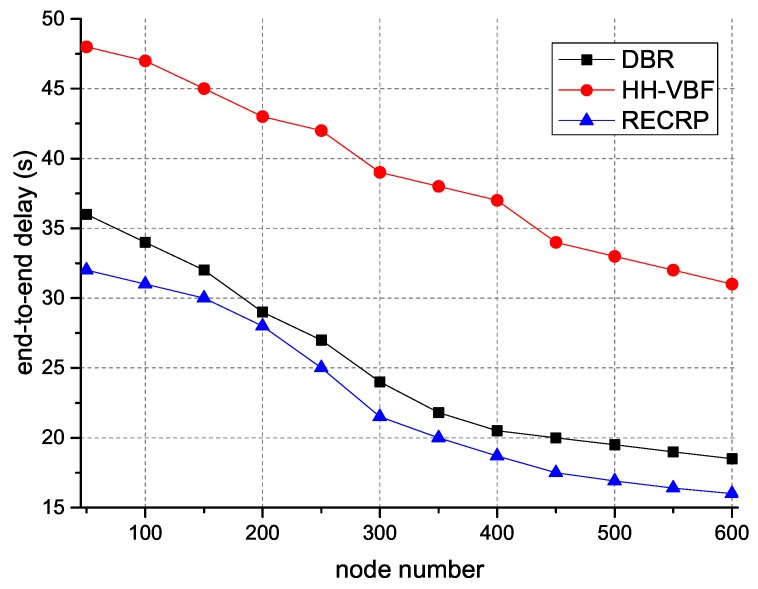
End-to-end delay with different numbers of nodes.

Figure 9 demonstrates the end-to-end delay with different numbers of nodes. This is because the retransmission times were reduced due to the high probability of successful delivery and the low probability of collisions. Thus, it is easy to observe that RECRP used less time to route packets from sensor nodes to sink nodes. This is because there were fewer alternative nodes for HH-VBF and the probability of successful delivery was reduced, resulting in extra retransmissions and longer end-to-end delay.

## 5. Conclusions

RECRP is an energy-efficient routing protocol, and it is suitable for long-term running applications such as underwater pollution detection and equipment monitoring (where real-time requirement is not desired). RECRP achieves a good performance for several reasons. Without extra hardware, we use RSSI and relative speed as double insurance to estimate the distance conservatively. As a single-copy routing protocol, we are able to achieve the lower overhead without redundant packets. Our max–min method not only dynamically changes power and frequency but also takes the two-hop forwarding capabilities into account to achieve energy efficiency. With the surface-to-bottom routing establishment mechanism, RECRP can handle communication voids. The simulation results demonstrated that RECRP significantly reduced the energy cost, packet loss ratio, and end-to-end delay compared with DBR and HH-VBF.

## Figures and Tables

**Table 1:** Routing table.

ID	Velocity	Distance	Energy	Level	Timer
1	0.2	1.3	30	1	12
3	0.1	0.8	15	3	17

**Table 2:** Ocean current variables.

Variable	Value	Variable	Value
*k*	1	$k_{1}$	N(0.001π,0.0001π)
$k_{4}$	0.015	$k_{2}$	N(0.01π,0.001π)
$k_{5}$	0.01	$k_{3}$	N(0.02π,0.002π)
λ	N(1,0.1)	*v*	N(0.1,0.01) m/s
